# In-Hospital Outcome of Patients with Diabetes Mellitus after CTO Recanalization with Third-Generation Drug-Eluting Stents

**Published:** 2019-04

**Authors:** Jan-Erik Guelker, Lars Bansemir, Rainer Ott, Thomas Rock, Rosemarie Guelker, Dong-In Shin, Heinrich Klues, Alexander Bufe

**Affiliations:** 1 *Department of Cardiology, Heart Centre Niederrhein, Helios Clinic Krefeld, Krefeld, Germany.*; 2 *Institute for Heart and Circulation Research, University Cologne, Cologne, Germany.*; 3 *RWI-Leibniz-Institute for Economic Research, Essen, Germany.*; 4 *Witten/Herdecke University, Witten, Germany.*

**Keywords:** *Coronary artery disease*, *Coronary occlusion*, *Diabetes mellitus*, *Treatment outcome*

## Abstract

**Background: **Percutaneous coronary intervention (PCI) of total chronic coronary occlusions (CTOs) still remains a major challenge in interventional cardiology. There is little knowledge in the literature about differences in CTO-PCI between diabetic and nondiabetic patients in the era of third-generation drug-eluting stents (DESs). In this study, we analyzed the impact of diabetes mellitus (DM) on procedural characteristics, complications, and acute outcomes in a cohort of 440 patients.

**Methods: **Between 2012 and 2016, we recruited 440 consecutive patients, 116 of them with DM. All the patients underwent PCI for at least 1 CTO. Antegrade and retrograde CTO recanalization techniques were applied. Only third-generation DESs were used. We used t-tests and the Pearson chi-quadrat test to test the significant differences in the variables between the 2 groups.

**Results: **The patients with DM were older than the nondiabetics (64.5 y vs. 61.1 y; P=0.003), and they suffered more frequently from a chronic kidney disease (7.1% vs. 2.4%; P=0.001). The nondiabetics less frequently had arterial hypertension (75.3% vs. 89.7%; P=0.001); however, they more often had a family liability for CAD (32.1% vs. 22.4%; P=0.050) and had a higher left ventricular ejection fraction (59.2% vs. 56.7%; P=0.011). The success rate was 85.2% in the patients without DM and 81.2% in the patients with DM (P=0.403). The existence of DM had no impact on the procedural success and complication rates.

**Conclusion: **Our study on 440 patients shows that diabetics and nondiabetics have similar success and complication rates after the recanalization of CTOs using third-generation DESs. It is a feasible and safe procedure and can be recommended as an alternative treatment.

## Introduction

The recanalization of chronic total occlusions (CTOs) remains a complex and challenging procedure in modern interventional cardiology. A CTO is defined as the duration of an occlusion lasting longer than 3 months and the presence of a thrombolysis in myocardial infarction (TIMI) flow grade 0 within the occluded segment.^1^ The prevalence of CTOs has been reported to be up to 30.0% among patients with a clinical indication for coronary angiography.^2^

Due to new interventional techniques and the use of further advanced sophisticated materials, the success rates of CTO recanalization have increased steadily in the past few years. In experienced hands, the reopening rates are more than 85.0%.^3^ The left ventricular function can be augmented, more invasive therapies such as a coronary artery bypass graft surgery (CABG) can be avoided at lower complication rates, and even the prognosis of the disease can be improved.^4^

Diabetes mellitus (DM) creates an inflammatory and atherothrombotic trait, accompanied by endothelial dysfunction.^5^ Patients with DM have an increased risk for cardiovascular complications such as myocardial infarction (MI), restenosis after percutaneous coronary intervention (PCI), and sudden cardiac death, particularly in CTO cases.^6^^-^^8^ Furthermore, the prevalence of CTOs is increased in patients with DM.^9^

For patients who suffer from a multivessel coronary artery disease and DM, CABG surgery is often considered to be the treatment of choice. The general outcome of patients with DM and CTOs treated with PCI is poorly investigated, and so far only a little knowledge exists about the acute clinical outcome following CTO-PCI in patients with DM.^10^

In this study, we evaluated the impact of DM on procedural characteristics, complications, and acute outcomes in a large cohort of patients between 2012 and 2016.

## Methods

A total of 440 patients were included in the heart center in Krefeld, Germany, between 2012 and 2016. The patients underwent PCI for at least 1 CTO. Diabetes mellitus was reported in 116 patients. 

The indications for inclusion were angina pectoris with a Canadian Cardiovascular Society (CCS) classification III and/or a positive functional ischemia test via magnetic resonance imaging or transthoracic echocardiography in the territory of the occluded artery of more than 10.0%. Both antegrade and retrograde CTO recanalization techniques were applied, and the procedures were performed in a standardized manner. 

Patients who were currently taking diabetes medications or had elevated levels of fasting and non-stressed blood glucose (>126 mg/dL) on at least 2 separate occasions during their hospital stay were defined as having DM.^11^^, ^^12^ The mean glycosylated hemoglobin (HbA1c) was 7.0% in the patients with DM.

To prevent thromboembolic complications, we administered heparin during the interventions guided by the activated clotting time (>250 s). All the procedures were performed via the arteria femoralis using 7-F guiding catheters. In the majority of the cases, bilateral injections of the contrast fluid were performed to determine the length of the lesion and the existence and extent of intercoronary collaterals. Only third-generation drug-eluting stents (DESs) were implanted. After PCI, a dual antiplatelet therapy consisting of 100 mg of aspirin once daily indefinitely and 75 mg of clopidogrel daily for at least 6 months was continued.

Procedural success was defined as a successful recanalization of the CTO and the restoration of TIMI flow grade 3. A composite safety end point summarizing severe complications such as all-cause mortality, vessel perforation, MI, and thromboembolic events was evaluated for each patient. Non–Q-wave MI was defined as an elevation of creatine kinase-MB to greater than 2 times the upper limit of normal with recurrent ischemic symptoms following CTO-PCI.^13^

An extra back-up (EBU) catheter was used for the left coronary artery, and either a JR4, an IMA, a Multipurpose, or an Amplatz catheter was applied for the right coronary artery. The selection of coronary guide wires followed a standardized concept of a step-up guide wire strategy starting with tapered polymer soft tip and ending up with super-stiff guide wires (12g wires). 

The continuous data are presented as means±standard deviations, and the categorical data are presented as numbers and percentages unless otherwise specified. We used *t*-tests and the Pearson chi-quadrat test to test the significant differences in the variables between the 2 groups. A P value of less than 0.05 was considered significant.

## Results

Table 1 shows the baseline characteristics of the patients with and without DM undergoing attempted CTO recanalization and the differences between the 2 groups with regard to age, sex, previous MI, hypertension, smoking habits, ejection fraction, previous CABG, chronic obstructive pulmonary disease, chronic kidney disease, and peripheral artery disease.

The patients with DM were older (64.5 y vs. 61.1 y; P=0.003), had a higher weight (88.9 kg vs. 85.3 kg; P=0.023), and more often had a chronic kidney disease (7.1% vs. 2.4%; P=0.001). In addition, there were fewer men (81.0% vs. 83.6%) and smokers (42.2% vs. 50.3%; P=0.136) among the diabetics than among the nondiabetics.

The nondiabetic patients less frequently had arterial hypertension (75.3% vs. 89.7%; P=0.001) and chronic obstructive pulmonary disease (7.1% vs. 15.5%; P=0.007); nonetheless, they more often had a familial liability for coronary artery disease (32.1% vs. 22.4%; P=0.050) and a higher ejection fraction (59.2% vs. 56.7%; P=0.011). The rate of patients with a peripheral artery disease and a prior CABG or MI was similar in both groups.

The procedural characteristics are presented in Table 2. Procedural success was independent of DM. The success rate was 85.2% in the patients without DM and 81.2% in the patients with DM; there was no significant difference between the 2 groups (P=0.403). 

**Figure 1:**
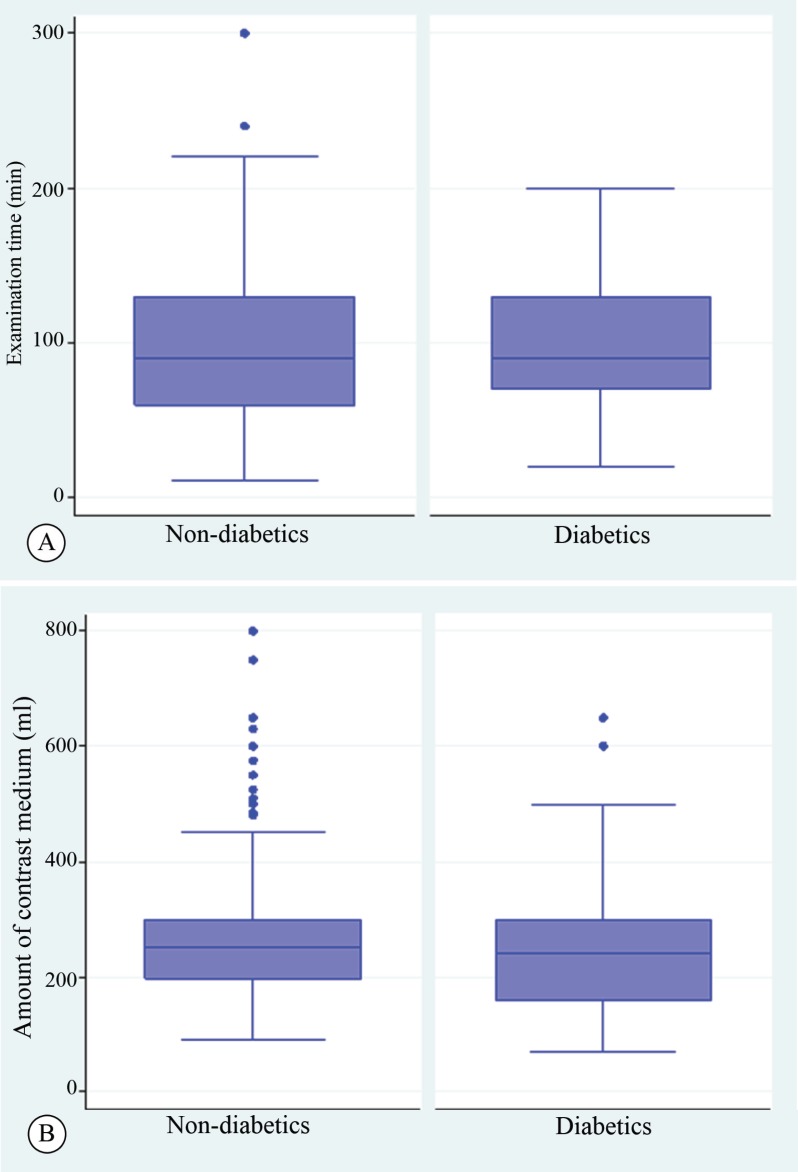
Comparisons of the diabetics and nondiabetics patients regarding examination time (A), and amount of the contrast medium (B).

The complication rate was low in both groups (2.6% vs. 1.9%; P=0.670), with no difference in statistical significance. The complications included mostly vascular complications such as hematoma and cardiac tamponade, which were successfully treated with a pericardiocentesis and without severe consequences. No differences were registered with respect to the amount of the contrast fluid (268.01±126.20 mL vs. 250.71±117.40 mL; P=0.198), the fluoroscopy time (36.20±22.08 min vs. 36.50±20.56 min; P=0.912), and the examination time (100.90±42.28 min vs. 98.40±44.59 min; P=0.596) as well as the number of stents and the stent diameters (Figure 1).

**Table 1:** Baseline characteristics of the paricipats*

	Diabetics (n=116)	Nondiabetics (n=324)	P
Age (y)	64.52±9.47	61.13±10.79	0.003
Male gender	94 (81.0)	271 (83.6)	0.522
Hypertension	104 (89.7)	244 (75.3)	0.001
Smoker	49 (42.2)	163 (50.3)	0.136
Family history for CAD	26 (22.4)	104 (32.1)	0.050
Chronic kidney disease	8 (7.1)	8 (2.4)	0.001
Prior MI	38 (32.8)	107 (33.0)	0.958
COPD	18 (15.5)	23 (7.1)	0.007
PAD	17 (14.7)	30 (9.3)	0.106
Prior CABG	11 (9.5)	27 (8.3)	0.705
Weight (kg)	88.92±14.26	85.33±15.04	0.023
Height (cm)	174.15±7.18	175.02±8.10	0.284
Ejection fraction (%)	56.77±9.13	59.24±8.85	0.011

CABG, Coronary artery bypass graft; CAD, Coronary artery disease; COPD, Chronic obstructive pulmonary disease; MI, Myocardial infarction; PAD, Peripheral artery disease

* Data are presented as mean±SD or n (%)

**Table 2:** Procedural characteristics*

	Diabetics (n=116)	Nondiabetics (n=324)	P
Single-vessel disease	25 (21.6)	85 (26.2)	0.318
Double-vessel disease	47 (40.5)	124 (38.3)	0.670
Triple-vessel disease	43 (37.1)	113 (35.0)	0.687
CTO in LAD	31 (26.7)	93 (28.7)	0.684
CTO in LCX	14 (12.1)	43 (13.3)	0.741
CTO in RCA	70 (60.3)	186 (57.7)	0.622
Blunt stump	42 (36.2)	123 (38.0)	0.737
Tortuosity of CTO	55 (47.4)	188 (58.0)	0.049
Retrograde approach	28 (24.1)	69 (21.3)	0.526
Complication rate	3 (2.6)	6 (1.9)	0.670
Success rate	95 (81.9)	276 (85.2)	0.403
Length of occlusion (mm)	39.24±16.44	40.05±16.78	0.673
Number of stents (n)	1.96±1.32	1.96±1.19	0.855
Diameter of stents (in mm)	3.01±0.34	3.12±0.37	0.379
Length of stents (mm)	65.31±27.74	65.78±27.27	0.903
Fluoroscopy time (min)	36.20±22.08	36.51±20.56	0.912
Amount of contrast medium (mL)	268.01±126.20	250.71±117.40	0.198
Examination time (min)	100.91±42.28	98.49±44.59	0.596
J-CTO Score	1.81±0.68	1.85±0.68	0.908

CABG, Coronary artery bypass graft; CAD, Coronary artery disease; COPD, Chronic obstructive pulmonary disease; J-CTO, Japanese CTO; MI, Myocardial infarction; PAD, Peripheral artery disease

* Data are presented as mean±SD or n (%)

## Discussion

DM is a highly prevalent medical condition globally and is frequently associated with symptomatic coronary artery disease, which needs to be treated with PCI. In the past, there was controversy regarding the choice of a DES in patients with DM.^14^

In our retrospective study, we compared the in-hospital outcome following the recanalization of CTOs with third-generation DESs between diabetic and nondiabetic patients. Our results showed no significant differences in the treatment results between the 2 groups.

Previous studies have demonstrated that the use of DESs, compared to bare-metal stents, is associated with a reduced need for repeat revascularization after CTO-PCI and that third-generation DESs are superior to first- or second-generation DESs with regard to several end points such as target-vessel failure, MI, and stent thrombosis in patients with DM. Recently, the TUXEDO-India trial showed that everolimus-eluting stents were superior to paclitaxel-eluting stents in this cohort.^14^

From earlier trials, it is known that in-hospital mortality following PCI may be increased in patients with DM when compared with those without DM.^15^ Sohrabi et al. reported a higher rate of in-hospital mortality and in-hospital major adverse cardiac events in diabetics (23.5% vs. 7.8%; P=0.021) after CTO-PCI.^13^ Furthermore, a higher rate of early revascularization within the first months was reported, corresponding to the fact that DM is an independent risk factor for restenosis.^5^

However, these results were obtained with bare-metal stents and first- and second-generation DESs. Higher rates of restenosis in diabetic patients were observed when there was an insufficient glycemic control (defined as HbA1c >7.0%).^12^ In our study, the mean HbA1c did not exceed 7.0%, which may have influenced the results.

Our data showed that these differences did not exist when third-generation DESs were applied. Particularly, the use of these new DESs accounts predominantly for our results since we know from a study by Werner et al. that in diabetic patients treated with bare-metal stents, the target-vessel failure rate was almost double that in nondiabetic patients (64.3% vs. 35.3%).^16^

Our data are in accordance with the results of a study conducted in 2011 by Claessen et al., who reported that procedural success rates were similar between the DM group and the non-DM group.^9^ Our results confirm these findings, show even a higher success rate (68.8% vs. 84.3%), and a halving of the contrast medium (473 mL vs. 259 mL) used for the procedures. This reflects the use of new techniques like the retrograde approach, new materials, particularly third-generation DESs, and routine bilateral injections during the procedure.

The incompleteness of revascularization is considered to be responsible for a decreased survival as shown by Safely et al., who demonstrated that patients with incomplete revascularization and severe multivessel coronary artery disease had a 5-year survival of 83.0% compared to 94.5% among those with complete revascularization (P<0.001).^17^

There are several limitations to our study. This is a retrospective study and all the data are collected from 1 single center. The results of this study may have been influenced by selection criteria, operator experience, and varying techniques used by the operators. Furthermore, there is no follow-up beyond the in-hospital phase and some data concerning cardiovascular risks like cholesterol or a prior stroke are not available. Another possible limitation is that the matched and unmatched data used in our study were already collected. Consequently, the analysis was based on an observational technique. Finally, that we did not use a multivariate analysis is another weakness of note.

## Conclusion

Our study, in accordance with previous studies, shows the technical feasibility of CTO-PCI in diabetics and nondiabetics and a comparable in-hospital outcome. CTO-PCI can be recommended as an interesting alternative strategy.

## Notes:

***This paper should be cited as: ***Guelker J. E, Bansemir L, Ott R, Rock T, Guelker R, Shin D. I, Klues H. G, Bufe A. In-Hospital Outcome of Patients with Diabetes Mellitus after CTO Recanalization with Third-Generation Drug-Eluting Stents. J Teh Univ Heart Ctr 2019;14(2):47-52.
